# COVID-19 and Fatal Sepsis Caused by Hypervirulent *Klebsiella pneumoniae*, Japan, 2020

**DOI:** 10.3201/eid2702.204662

**Published:** 2021-02

**Authors:** Tomohiro Hosoda, Sohei Harada, Koh Okamoto, Sumire Ishino, Makoto Kaneko, Masahiro Suzuki, Ryota Ito, Miyuki Mizoguchi

**Affiliations:** Kawasaki Municipal Kawasaki Hospital, Kanagawa, Japan (T. Hosoda, S. Ishino, M. Kaneko);; The University of Tokyo Hospital, Tokyo, Japan (S. Harada, K. Okamoto, M. Mizoguchi);; Fujita Health University School of Medicine, Aichi, Japan (M. Suzuki, R. Ito)

**Keywords:** 2019 novel coronavirus disease, coronavirus disease, COVID-19, severe acute respiratory syndrome coronavirus 2, SARS-CoV-2, viruses, respiratory infections, zoonoses, hypervirulent *Klebsiella pneumoniae*, bacteria, co-infection, sepsis, string test, Japan

## Abstract

A patient in Japan with coronavirus disease and hypervirulent *Klebsiella pneumoniae* K2 sequence type 86 infection died of respiratory failure. Bacterial and fungal co-infections caused by region-endemic pathogens, including hypervirulent *K. pneumoniae* in eastern Asia, should be included in the differential diagnosis of coronavirus disease patients with acutely deteriorating condition.

For a minority of patients, bacterial and fungal co-infections can complicate the course of coronavirus disease (COVID-19) (*1*,*2*). Co-infection can contribute to the poor prognosis for patients with COVID-19, especially for high-risk populations such as elderly patients (*3*). Indeed, a large retrospective multicenter study reported that for half of the patients who died of COVID-19, secondary bacterial co-infection developed during hospitalization (*3*). In a retrospective study in China, the second most common respiratory pathogen detected from patients with COVID-19 was *Klebsiella pneumoniae*, following only *Streptococcus pneumoniae* (*4*).

Hypervirulent *K. pneumoniae* (hvKp) was originally recognized as a pathogen that causes severe community-acquired infections among relatively healthy persons. hvKp isolates carry virulence plasmids that harbor cardinal virulence genes, and with higher frequency than classical *K. pneumoniae* they cause disseminated infections involving liver, lungs, central nervous system, and eyes (*5*,*6*). Although hvKp infections have been reported mainly from hvKp-endemic areas such as eastern Asia, in recent years, sporadic cases have been increasingly reported worldwide (*7*). Furthermore, recent studies from hvKp-endemic areas demonstrated that hvKp is often associated with healthcare and hospitalization for elderly and debilitated populations (*8*,*9*). A multicenter study in Japan showed that more than half of bloodstream infections caused by hvKp occurred as healthcare-associated or hospital-acquired infections (*8*).

Therefore, hvKp infections may have the potential for seriously complicating the course of COVID-19, especially in hvKp-endemic areas. We describe a fatal case of superimposed hvKp infection in an elderly woman with COVID-19 in Japan.

## The Case

In August 2020, an 87-year-old woman sought care at an emergency department for a 4-day history of fever and dry cough. The day before, COVID-19 had been diagnosed for 2 family members living with her. The woman had hypertension, dyslipidemia, and dementia and had been receiving outpatient care at a nursing home 5 days a week. At admission, her vital signs were temperature 37.7°C, blood pressure 202/93 mm Hg, pulse rate 61 beats/min, respiratory rate 16 breaths/min, and oxygen saturation 95% while breathing ambient air. Physical examination findings were otherwise unremarkable. Laboratory studies revealed 2,660 leukocytes/μL, including 811 lymphocytes/μL; 13.8 × 10^4^ platelets/μL; aspartic aminotransferase 36 U/L; alanine transaminase 22 U/L; creatinine 0.81 mg/dL; blood glucose 83 mg/dL; and ferritin 268.2 ng/mL. Coagulation studies showed elevated D-dimer of 0.8 μg/mL with prothrombin time or activated partial thromboplastin time within normal range. COVID-19 was diagnosed on the basis of a positive COVID-19 rapid antigen test result (ESPLINE SARS-CoV-2; Fujirebio Diagnostics, https://www.fujirebio.com). Shortly after admission, the patient became hypoxic (oxygen saturation 89% while breathing ambient air) and required supplemental oxygen delivered by nasal cannula at 2 L/min. 

**Figure 1:**
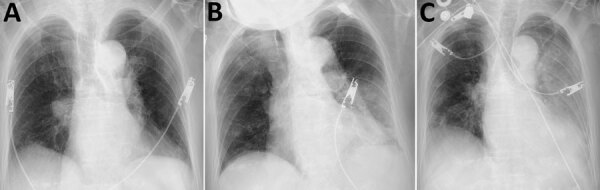
Chest radiographs (anteroposterior views) of hospitalized patient with coronavirus disease and fatal superimposed hypervirulent *Klebsiella pneumoniae* K2 sequence type 86 infection, Japan, 2020. A) Hospitalization day 1 (admission), showing no ground glass opacity and consolidation. B) Hospitalization day 7, showing asymmetric infiltrates with pleural effusion, mainly in left lung. C) Hospitalization day 8, showing infiltrate spread to right lower lung and worsened pleural effusion in left lung.

On hospitalization day 2, a chest radiograph showed no infiltrates (Figure 1, panel A); dexamethasone (6 mg/d) was initiated out of concern for hypoxia from COVID-19. Over the next 2 days, fever and dry cough subsided, and hypoxia gradually improved to an oxygen saturation of 96% while breathing ambient air. On hospitalization day 7, she experienced fever with productive cough and hypoxia (oxygen saturation of 90% while breathing supplemental oxygen at 6 L/min through a nonrebreathing oxygen mask). A chest radiograph revealed infiltrates in the left lung with pleural effusion (Figure 1, panel B). Ampicillin/sulbactam was started. On hospital day 8, her condition rapidly deteriorated; hypoxia and the lung infiltrates in the left lung worsened (Figure 1, panel C). The antimicrobial drug was switched to piperacillin/tazobactam. The patient and her family did not request escalation of her care to intensive care, which would have included mechanical ventilation; on hospitalization day 9, she died of respiratory failure.

**Figure 2:**
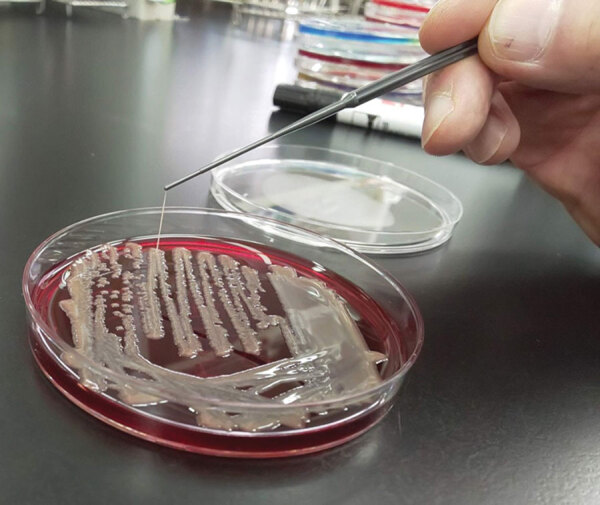
Positive string test result for *Klebsiella pneumoniae* isolate from blood of patient with coronavirus disease and fatal superimposed hypervirulent *Klebsiella pneumoniae* K2 sequence type 86 infection, Japan, 2020.

Sputum and blood collected for culture on hospitalization day 7, along with sputum collected for culture on the day of admission, grew *K. pneumoniae*. All 3 isolates were positive by string test (showed viscous strings >5 mm when stretched with a standard inoculation rod) (Figure 2) and were susceptible to all antimicrobial drugs tested except ampicillin. We analyzed the virulence gene profiles of these isolates by using multiplex PCR as described previously (*10*), and we identified carriage of genes for capsular genotype K2, *iutA*, *rmpA*, *entB*, *mrkD*, and *ybtS*. Multilocus sequence typing with standardized protocol demonstrated that these isolates belonged to sequence type (ST) 86 (*11*). We further analyzed the isolate from blood (FUJ01174) with whole-genome sequencing by using Miseq (Illumina, https://www.illumina.com) as described previously (*8*), and we confirmed carriage of virulence genes *rmpA*, *rmpA2*, *iroBCDN*, *irp1*, *iucABCD*, *iutA*, *ybtARPQSTUX*, *kvgAS*, *fyuA*, and *mrkABDFHIJ* by using the *Klebsiella* locus/sequence definitions database (https://bigsdb.pasteur.fr/klebsiella). In addition, we identified *Peg-344* with a manual BLASTn (https://blast.ncbi.nlm.nih.gov) search (reference sequence, GenBank accession no. AP006726). Assembled contigs covered the nucleotide sequence of pLVPK (GenBank accession no. AY378100), a prototypical *K. pneumoniae* virulence plasmid, with 91.8% coverage and 99.9% identity (Appendix). We deposited genomic sequences of the FUJ01174 strain in the National Center for Biotechnology Information database under BioSample accession no. SAMN16787939.

## Conclusions

For this COVID-19 patient who died of superimposed *K. pneumoniae* infection, the causative strain recovered from blood and sputum belonged to K2-ST86, a prototypical hvKp, together with K1-ST23. Furthermore, the isolate carried the cardinal hvKp virulence genes *rmpA*, *rmpA2*, *iroBCDN*, *iucABCD*, and *peg-344*, which have been recognized as molecular markers for the identification of hvKp that carry high risk for disseminated and fatal infections (*6*,*8*).

This case highlights 2 implications for the management of COVID-19 patients. First, bacterial and fungal co-infection may occur relatively early in the course of COVID-19. The condition of the patient reported here rapidly deteriorated 10 days after symptom onset; she had initially recovered after admission and treatment with dexamethasone. Although the timing (10 days after symptom onset) was typical for acute respiratory distress syndrome and acute cardiac injury resulting from COVID-19 itself (*12*), this patient instead experienced a fatal bacterial infection. Given the low prevalence of bacterial co-infections among COVID-19 patients, judicious use of antimicrobial drugs is recommended (*13*). However, this case emphasizes that timely antimicrobial treatment is crucial for patients with suspected or confirmed bacterial co-infection. Furthermore, corticosteroid treatment for COVID-19 may increase the risk for and severity of bacterial co-infection. Therefore, consideration for empiric antimicrobial therapy and thorough evaluation for bacterial co-infection should be considered for COVID-19 patients with acutely deteriorating condition. Second, local epidemiology should be considered when presuming a causative pathogen for patients with bacterial and fungal co-infections (*14*). Prevalence of hvKp infection in eastern Asia is exceptionally high (*8*). It is possible that a substantial number of superimposed hvKp infections complicating COVID-19 may have been unrecognized because the microbiological criteria for diagnosing hvKp widely used at microbiology laboratories in healthcare facilities (identifying carriage of genes for capsular genotype and string test) may not have been routinely available. For the case we report, respiratory colonization of hypermucoviscous *K. pneumoniae* was noted on culture at admission. Because colonization by hvKp is an established risk factor for subsequent hvKp invasive disease (*15*), additional caution is required for superimposed hvKp infections when caring for COVID-19 patients known to be colonized with hvKp.

In conclusion, we report a fatal case of hvKp infection superimposed on a patient with COVID-19. When the condition of COVID-19 patients worsens, bacterial and fungal infections, including region-endemic infections (hvKP in eastern Asia), should be included as a differential diagnosis and require appropriate evaluation and treatment in a timely fashion. 

Appendix Contigs from isolate from blood of patient with coronavirus disease and fatal superimposed hypervirulent *Klebsiella pneumoniae* K2-ST86 infection, Japan, compared with contig of pLVPK.
