# Supplementary material for: COVID-19 and Fatal Sepsis Caused by Hypervirulent Klebsiella pneumoniae, Japan, 2020
Source: Emerg Infect Dis. 2021 Feb;27(2):556–9. doi: 10.3201/eid2702.204662 (PMC7853555; doi:10.3201/eid2702.204662)
Supplement: Appendix — Contigs from isolate from blood of patient with coronavirus disease and fatal superimposed hypervirulent Klebsiella pneumoniae K2-ST86 infection, Japan, compared with contig of pLVPK. [file 20-4662-Techapp-s1.pdf]

Article DOI: https://doi.org/10.3201/eid2702.204662

COVID-19 and Fatal Sepsis Caused by Hypervirulent *Klebsiella pneumoniae*, Japan, 2020

Appendix

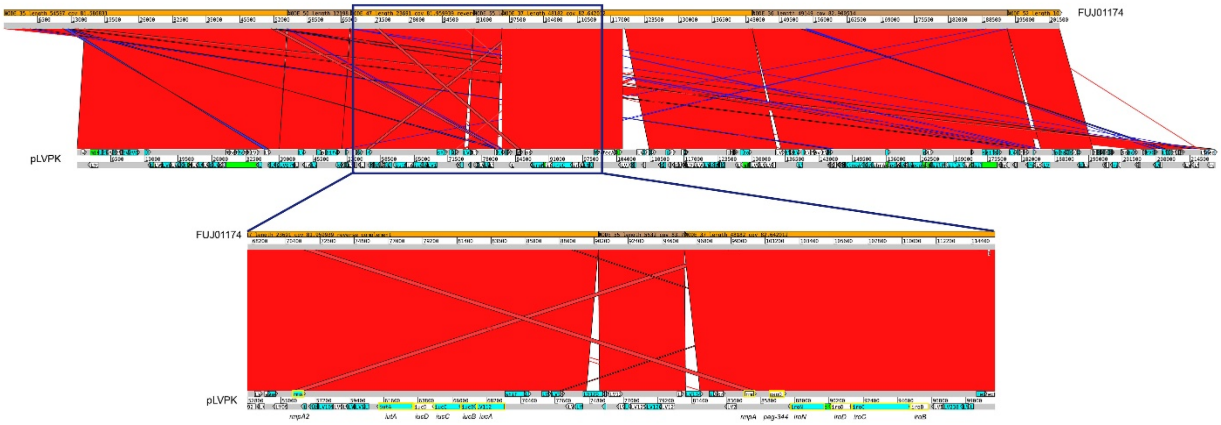

Appendix Figure. Contigs of FUJ01174 with high similarity to the nucleotide sequences of pLVPK plasmid (AY378100.1) were identified with BLASTn search. Pairwise comparison of contigs of FUJ01174 and pLVPK was performed with Artemis Comparison Tool (https://www.sanger.ac.uk/tool/artemis-comparison-tool-act).
